# A New Modified Artificial Bee Colony Algorithm with Exponential Function Adaptive Steps

**DOI:** 10.1155/2016/9820294

**Published:** 2016-05-17

**Authors:** Wei Mao, Heng-you Lan, Hao-ru Li

**Affiliations:** ^1^Department of Mathematics, Sichuan University of Science & Engineering, Zigong, Sichuan 643000, China; ^2^Key Laboratory of Higher Education of Sichuan Province for Enterprise Informationalization and Internet of Things, Zigong, Sichuan 643000, China; ^3^School of Automation and Electronic Information, Sichuan University of Science & Engineering, Zigong, Sichuan 643000, China

## Abstract

As one of the most recent popular swarm intelligence techniques, artificial bee colony algorithm is poor at exploitation and has some defects such as slow search speed, poor population diversity, the stagnation in the working process, and being trapped into the local optimal solution. The purpose of this paper is to develop a new modified artificial bee colony algorithm in view of the initial population structure, subpopulation groups, step updating, and population elimination. Further, depending on opposition-based learning theory and the new modified algorithms, an improved *S*-type grouping method is proposed and the original way of roulette wheel selection is substituted through sensitivity-pheromone way. Then, an adaptive step with exponential functions is designed for replacing the original random step. Finally, based on the new test function versions CEC13, six benchmark functions with the dimensions *D* = 20 and *D* = 40 are chosen and applied in the experiments for analyzing and comparing the iteration speed and accuracy of the new modified algorithms. The experimental results show that the new modified algorithm has faster and more stable searching and can quickly increase poor population diversity and bring out the global optimal solutions.

## 1. Introduction

It is well known that algorithms for solving various characteristics optimization problems can be classified into different groups, such as population-based algorithms, stochastic algorithms, deterministic algorithms, and iterative algorithms. An algorithm is called population-based [1] if one works with a group of solutions and tries to improve them. Two important classes of population-based optimization algorithms are exactly evolutionary algorithms and swarm intelligence-based algorithms [2]. Swarm intelligence is an innovative artificial intelligence technique with collective behavior of self-organized systems [3]. Since many swarm intelligence algorithms, such as genetic algorithm (GA) [4], particle swarm optimization (PSO) [5], ant colony optimization (ACO) [6], and biogeography-based optimization [7], have simplicity, ease of implementation, outstanding performance, and other advantages [8], they have shown great success in solving some nonconvex, discontinuous, or nondifferentiable optimization problems. However, these intelligence algorithms are sensitive to value and precision. Thus, inspired by the behavior of honey bees, Karaboga [9] introduced basic artificial bee colony algorithm (ABC) in 2005 and constituted one of the most prominent approaches in the field of bee-inspired algorithms. Further, in consideration of the solution reaching speed, the success rate, and the performance rate, El-Abd [10] provided a complete performance assessment of ABC and compared it with the widely known differential evolution (DE), GA, heuristic algorithms, PSO, and other foraging algorithms (e.g., bacterial algorithm, ACO, and bacterial foraging optimization) by using the well-known benchmark functions in [11].

It was claimed that ABC is the most successful algorithm for multimodal and hybrid functions [12]. This is because ABC has no demand to the objective function, constraint, and external information and is only based on fitness function in the search process [13]. Further, ABC has the following advantages. (i) The mechanism of multiple roles: using different methods, bees adjust quality of the solutions spontaneously, so as to adapt to the next search process [14]. (ii) The cooperative working mechanism: according to the information from other bees, bees decide whether to find the optimal solution with larger probability [15]. (iii) The strong robustness: the search rules are not certain but are probabilistic and have excellent robustness and a wide range of applicability [16]. (iv) The stability: even if the individual fails, the entire swarm can still complete the task [17]. (v) Less control parameters, simple operation, and ease of implementation [18]: indeed, ABC has been shown to be very competitive with respect to other state-of-the-art foraging and evolutionary algorithms.

However, there exists still an insufficiency to ABC because ABC does well in exploration but is poor at exploitation. As for the improvement and development of ABC, Karaboga and Gorkemli [19] proposed a new update rule for onlooker bees in the hive to improve the local search and convergence characteristics of ABC. Inspired by PSO, Imanian et al. [20] changed the update rule of basic ABC to increase the convergence speed for solving high dimensional and continuous optimization problems. Wang et al. [21] proposed multistrategy ensemble artificial bee colony algorithm (MEABC) to improve the local and global search capability of basic ABC and tested the performance of MEABC by using basic, shifted, and rotated benchmark functions. Gao et al. [22] developed new search equations to adjust exploration and exploitation capability of ABC. In a different approach for ABC, Das et al. [23] proposed a learning routine based on fitness and proximity stimuli and tested the method with standard benchmark functions. Zang et al. [24] designed a logarithmic function adaptive step instead of the original random step.

Moreover, in dealing with some complex problems by applying ABC, there are some defects such as slow search speed, poor population diversity, stagnation in the working process, and trapping into the local optimal solution [13]. Recently, ABC has been extended and improved by many researches. But since ABC is relatively new, the researches in the literatures lack systematicness and are scattered. See, for example, [1–3, 12–30] and the references therein. In 2012, Li et al. [31] pointed out that “ABC has no mechanism to use the global information in the search space, so it easily results in a waste of computing power and gets trapped in local optima”. Further, the authors proposed a novel algorithm (named as DEABC, i.e., differential evolution artificial bee colony algorithm), which synthesizes DE and ABC and enhances individuals by sharing information between DE population and bee colony. For related works, one can see [1, 30] and the references therein.

Motivated and inspired by the above works, a new modified artificial bee colony (MABC) algorithm shall be constructed based on adaptive step with exponential functions. By using opposition-based learning theory and an improved *S*-type grouping method, the initial population for MABC will be given, and the original way of roulette wheel selection shall be substituted by sensitivity-pheromone way. Specifically, an adaptive step with exponential functions will be designed to replace the original random step. In order to verify the validity of the improved algorithm, MABC, it is compared with DEABC [30], novel artificial bee colony algorithm (NABC) [8], and ABC; the experiment results tested with six well-known benchmark optimization functions, which are chosen from new test function versions CEC13, show that MABC is superior to ABC, NABC, and DEABC.

The rest of this paper is organized as follows.  Section 2 gives a brief introduction to ABC. The new MABC is presented and analyzed in Section 3.  Section 4 presents and discusses the experimental results of six benchmark functions with the dimensions *D* = 20 and *D* = 40, respectively. Finally, the conclusion is drawn in Section 5.

## 2. Brief Review to ABC

In this section, a brief review on ABC is going to be given.

### 2.1. Thoughts of the Algorithm

A honey bee colony can successfully discover the highest quality food sources in nature. Hence, the idea of ABC comes from intelligent foraging behavior of honey bees to finding good solutions for solving optimization problems. In a general way, according to the ways of searching food, the colony of bees is divided into three kinds: employed bees, onlooker bees, and scout bees. The employed bees are responsible for exploiting the nectar sources. They explore the beforehand food source position and give the quality information of the food to the onlooker bees. The onlooker bees wait in the hive and decide to exploit a food source based on the information shared by the employed bees. In order to find a new nectar source, the scout bees randomly search environment either depending on an internal motivation or based on possible external clues [32]. The position of a nectar source implies a possible solution of the optimization problems, and the profitability of a nectar source corresponds to the quality (fitness) of the possible solution. Each nectar source is exploited by only one employed bee. In other words, the number of nectar sources equals the number of employed bees or onlooker bees [25]. In this process, the employed bees maintain good solution, the onlooker bees improve convergence speed, and the scout bees enhance the ability to remove local optimum [26, 31].

### 2.2. ABC Iteration Steps

From [24, 33], it follows that main iteration steps of ABC can be listed as follows.

**Step 1 (initialization).**

Randomly generate *N* solutions (i.e., food sources) {*x*_1_, *x*_2_,…, *x*_*N*_} as initial population in a *D* dimension searching space, where *N* is the number of food sources, which equals the half of the colony size, *x*_*i*_ = (*x*_*i*1_, *x*_*i*2_,…, *x*_*iD*_) is a *D*-dimensional solution vector and the *i*th food source in the initial population for *i* = 1,2,…, *N*, and *D* also denotes the number of optimization parameters.

**Step 2 (renewing population).**

In the stage of collecting honey, each employed bee produces a new nectar source within the neighborhood of the food source. After comparing the new nectar source with the old ones, the high probability will be memorized. Next, in the stage of follow, every onlooker bee evaluates the profitability of nectar sources taken from all employed bees and then chooses a food source at a certain probability. As in the case of the employed bees, she produces a modification on the source position in her memory and keeps the better nectar source. The regeneration of nectar sources in these two stages is based on the following formula:

(1) $$\begin{array}{c} \nu_{ij}=x_{ij}+\mathrm{rand}\cdot \left(\;x_{ij}-x_{kj}\;\right), \end{array}$$

where *i*, *k* = 1,2,…, *N*, *j* = 1,2,…, *D*, and rand ∈ [0,1] is a random number, which controls the generation range of neighborhood of *x*_*ij*_. With the search being close to optimal solution, the range of neighborhood will become smaller and smaller.

**Step 3 (nectar source selection).**

In the stage of follow, the onlooker bees choose food source through comparing the probability which is computed by the fitness value. The nectar sources of high probability are selected in large probability. And the probability of being selected for the food sources is calculated as follows:

(2) $$\begin{array}{c} P_{i}=\frac{{\text{Fit}}_{i}}{\sum_{i=1}^{N}{\text{Fit}}_{i}}, \end{array}$$

where Fit_*i*_  (*i* = 1,2,…, *N*) is the fitness (profitability) value of the *i*th solution, which is obtained by the following equation:

(3) $$\begin{array}{c} {\text{Fit}}_{i}=\left\{\begin{array}{cc} \frac{1}{1+f_{i}}, & \text{if }f_{i}\geq 0,\\ 1+\left|f_{i}\right|, & \text{if }f_{i}<0, \end{array}\right. \end{array}$$

where *f*_*i*_ is the objective function value for *i* = 1,2,…, *N*, which is specific for the optimization problem. If the new food source position has a quality equal to or better than the old one, then the old food source position is replaced by the new one. Otherwise, the old one is retained, which is the same as the employed bees stage.

**Step 4 (population elimination).**

If a solution has not been improved significantly through a predetermined number of trials, called “max iteration,” then the solution is regarded as falling into a local optimal solution and their original position will be abandoned. Thus, the corresponding employed bees will become scout bees and a new solution instead of the eliminated solution is randomly generated, which can be expressed as follows:

(4) $$\begin{array}{c} x_{ij}=\mathrm{rand}\cdot \left(\;x_{\max j}-x_{\min j}\;\right)+x_{\min j}, \end{array}$$

where *i*, *j* are the same as in (1) and *x*_max*j*_ and *x*_min*j*_ denote the *j*th individual maximum and the *i*th individual minimum values, respectively.

Based on the above iteration steps, the process of ABC [12] can be shown in Algorithm 1.

## 3. New MABC

From the above introductions, it follows that ABC has many advantages, such as simplicity, ease of implementation, and outstanding performance. However, the algorithm steps indicate that ABC has the following obvious flaws:

- The randomly generated initial population will lead the solution to be random distribution in the solution space. Hence, the searching ability of ABC will be affected directly.
- All individuals begin to search directly in the whole solution space, which will reduce searching efficiency.
- The population is regenerated by random step length. When the algorithm performs in the neighborhood of optimal solutions, the searching range will be severely restricted. Thus, the convergence rate and optimization precision of ABC will be influenced.

In order to overcome the existing deficient problem of ABC and related algorithms, the purpose of this paper is to introduce and study a new MABC and to improve the initial population structure, the population grouping, and the population regeneration of ABC.

### 3.1. Opposition-Based Learning of Population Initialization

ABC is relatively sensitive to the initial population construction. Therefore, the search range of ABC will be restricted and the global search ability of ABC will also be influenced when the initial population distribution in the search space or the local area is random. However, the initial population constructed by using opposition-based learning method has the diversity as much as possible, is better representative, and meets the accuracy of experimental requirements (see, e.g., [6, 13, 34, 35] and the references therein).

Based on the opposition-based learning method, the initial population for MABC will be constructed in this paper. Firstly, an initial solution is given, and next the corresponding reverse initial solution to the initial solution is produced. Then, the two kinds of solutions are sorted. Finally, the better fitness solution is chosen as the initial population. This method will help to enhance the efficiency of MABC and to improve the quality of solutions [34]. The processes can be listed as shown in Algorithm 2.

### 3.2. *S*-Type Subpopulation Grouping

Intelligent optimization algorithms commonly use general grouping method as follows. Firstly, the population fitness function value of each individual is calculated. Secondly, the fitness function value is arranged in descending order. Then, the entire ordered population is divided into *M* subpopulations, and the process of the grouping [24] is as shown in Table 1. However, this grouping method still causes some problems: the relative advantage of individuals is divided into the same species and their disadvantage is concentrated in other uncertain groups. This phenomenon will make very large differences between subpopulations, and this grouping method is not conducive to the expansion of population diversity.

To reduce the difference between each group (see [24]), a new grouping method is presented, which is called *S*-type subpopulation grouping. Similarly, the fitness function value is arranged in descending order. But, relative to the general grouping method, the order of the value in the new groups is different, and the process of new grouping is listed in Table 2.

Indeed, first of all, the fitness function values of each individual of population are calculated, and the fitness function values in descending order are ranked. Next, the entire population is divided into *M* subpopulations *S*_1_, *S*_2_,…, *S*_*M*_. Let *Y*_*i*_ be the *i*th individual for *i* = 1,2,…, *N*. If the solutions due to *S*-type subpopulation grouping are odd rounds and there are a large number subpopulations, then there will be more differences between each group. Thus, the final round can be abandoned through *S*-type subpopulation grouping. Now one can see that *S*-type subpopulation grouping can overcome the disequilibrium between groups, making the subpopulation have a little difference, which is good to the expansion of population diversity.

Furthermore, a new population will be recomposed by all subpopulations when each subpopulation group is less than max iteration *N* of intragroup. And then the fitness function values of each population are recalculated and are arranged according to the descending order. Only one of the individuals of equivalent fitness function values is kept. Let the number of remaining individuals at last be *K*. Then the population can be regenerated as follows:

(5) the top M individuals are retained,if K≥M,M-K individuals will randomly be initialized and filled into the population,if K<M.

### 3.3. Sensitivity-Pheromone Option

Since the onlooker bees chosen by roulette way are too greedy and the population diversity is deduced too quickly, it is easily plunged into local optimum by using ABC for solving function optimization problems. In applications, the roulette way is practically affected by the population diversity.

Thus, it is an important idea for sensitivity to replace the roulette way. Sensitivity is put forward by free search algorithm [35] with pheromone (related to optimization of adaptive value) to selected area, which can be expressed as follows:

(i) Calculate the *N* values of fitness to the individual *f*(*x*).
(ii) For each *i* = 1,2,…, *N*, calculate the *i*th pheromone
  (6) $$\begin{array}{c} p\left(\;i\;\right)=\frac{a\cdot f\left(i\right)}{f_{max}}+\left(\;1-a\;\right),\;a=\frac{f_{max}}{\sum_{i=1}^{N}f_{i}}, \end{array}$$
 where *f*_max_ stands for the maximum values of *f*(*i*) for *i* = 1,2,…, *N*.
(iii) Generate the *j*th individual sensitivity randomly: *S*(*j*) ~ *U*(0,1).
(iv) Find out sensitivity area *i* matching individual of the first *j* and randomly find *i* to meet *p*(*i*) ≥ *S*(*i*).

By the above model, it is easy to see the following: (i) since the sensitivity-pheromone is random, any area can be searched in theory, and the local optimum can largely be avoided by using the model. (ii) The fourth step of the model is to make MABC have a clear direction, and convergence or divergence of the objective function in the search space can be determined quickly. This method is similar to the roulette way of nectar source for the onlooker bees, and so sensitivity and pheromone option instead of roulette way can be applied.

### 3.4. Updating Step Length with Exponential Function

According to solution search equation (1) of ABC, the new candidate solution is generated through moving the old one towards another selected random solution of the population. This method can increase the probability of locating global optimal solution, but it cannot guarantee obtaining a better solution. Hence, the convergence speed is slow. If each new candidate solution is better than the old one, then convergence speed will become faster. However, if only the better solution is considered, then the algorithm could get trapped into local optimum [32]. In ABC, the updating step length denoted by *σ* (i.e., rand in (1)) plays a very important role and controls the ability to search (local or global) and the convergence rate. On the other hand, if the value of *σ* is too large, then the algorithm is easy to lose the global optimal solution. And, on the contrary, the algorithm is easy to show the phenomenon of stagnation and fall into local optimum [33]. Therefore, it is very necessary to design a new suitable step length associated with the performance of the algorithm.

The following exponential function is used in this paper to update step length:

(7) $$\begin{array}{c} \sigma =\left(\;\mathrm{rand}-0.5\;\right)\cdot 2^{\mathrm{rand}}, \end{array}$$

and the improved population moving step length is

(8) $$\begin{array}{c} \Delta =\sigma \cdot \left(\;x_{ij}-x_{kj}\;\right)=\left(\;\mathrm{rand}-0.5\;\right)\cdot 2^{\mathrm{rand}}\cdot \left(\;x_{ij}-x_{kj}\;\right), \end{array}$$

for *i*, *k* = 1,2,…, *N* and *j* = 1,2,…, *D*, where rand⁡ is the same as in (1). Thus, the location of the updated solution is

(9) νijxij+σ·xij−xkj=xij+rand⁡−0.5·2rand⁡·xij−xkj

and the location of the random generated solutions is

(10) $$\begin{array}{c} x_{ij}=x_{\min j}+\left(\;\mathrm{rand}-0.5\;\right)\cdot 2^{\mathrm{rand}}\cdot \left(\;x_{\max j}-x_{\min j}\;\right), \end{array}$$

where *x*_max*j*_ and *x*_min*i*_ are the same as in (*∗*).

Now one can know that with the increase of evolution algebra, the updating step will be increased and become larger and larger. In the early stage of the evolution, the improved evolution algebra can be conducted a comprehensive local search, and make the algorithm better position to the target area. In the middle and later periods of the evolution, the improved evolution algebra will be carried out global search and can speed up the convergence rate so as to reach to optimal solution.

### 3.5. Flowchart of MABC

To clearly know MABC, the detailed flowchart of MABC is shown in Algorithm 3, where the max iteration as the termination criteria is adopted the same as in Algorithm 1, and CS denotes the number of colony size, which is equal to the sum of the numbers of employed bees and onlooker bees.

## 4. Simulation and Analysis

In this section, the performance and accuracy of MABC, ABC, NABC, and DEABC will be analyzed and compared through the figures, the total run time, mean value, and standard deviation of six benchmark functions from the new test function versions CEC13, respectively.

### 4.1. Convergence Performance Analysis

In order to verify the validity of MABC, six benchmark functions listed in Table 3 (see [11, 27]) are used in the experiments. According to their characteristics, these six functions are divided into two groups. The Sphere, SumSquares, and Schwefel 2.22 are unimodal functions, and Ackley, Griewank, and Rastrigin are multimodal functions. For each benchmark function, two dimension sizes, 20 and 40, of solution space are tested. The colony size is 80 and the max iteration is 1500; limit is 100. Each of the experiments is repeated 10 times.

**Figure 1:**
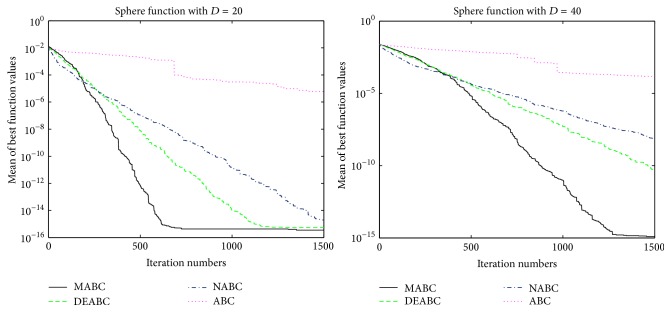
Convergence performance of ABC, DEABC, NABC, and MABC.

**Figure 2 fig2:**
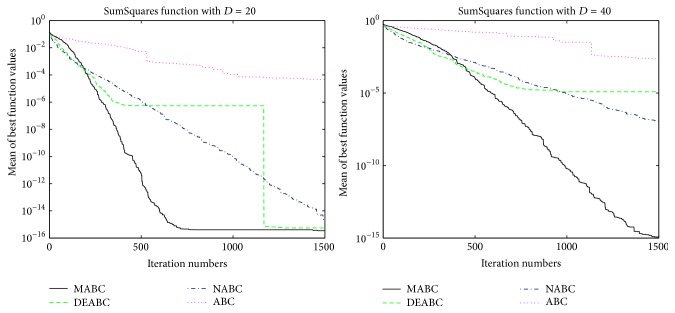
Convergence performance of ABC, DEABC, NABC, and MABC.

**Figure 3 fig3:**
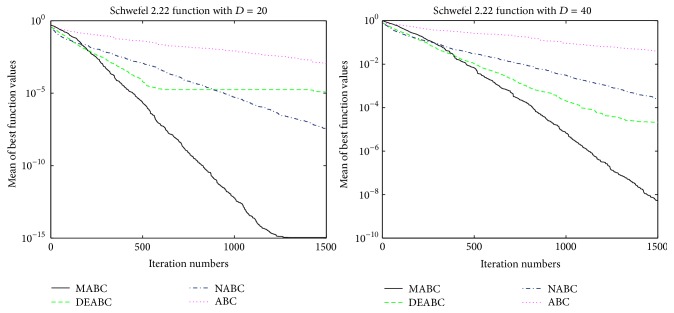
Convergence performance of ABC, DEABC, NABC, and MABC.

**Figure 4 fig4:**
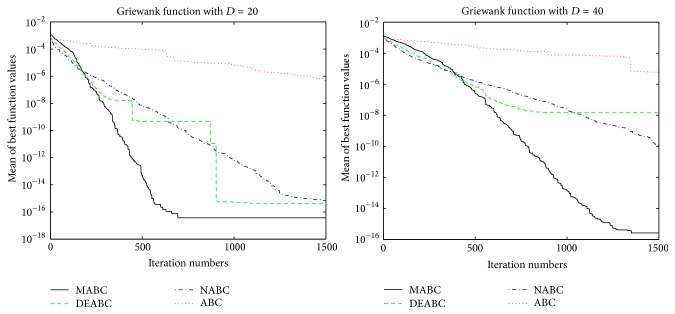
Convergence performance of ABC, DEABC, NABC, and MABC.

**Figure 5 fig5:**
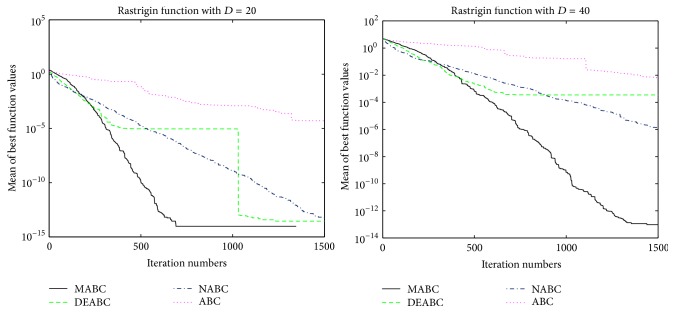
Convergence performance of ABC, DEABC, NABC, and MABC.

**Figure 6 fig6:**
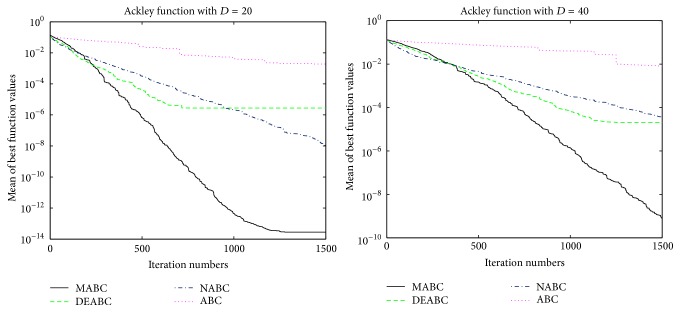
Convergence performance of ABC, DEABC, NABC, and MABC.

In order to further compare the performance of four proposed algorithms, convergence performance of the six functions for ABC, DEABC, NABC, and MABC is plotted to dimension sizes 20 and 40, respectively. See Figures 1 [Fig fig2] [Fig fig3] [Fig fig4] [Fig fig5]–6.

From the convergence performances of SumSquares, Griewank, and Rastrigin functions, one can see that when *D* = 20, ABC converges with the slowest speed and locates a local optimal solution. DEABC has the fastest speed at the beginning and goes into the global optimal solution. NABC also goes into the global optimal solution, but the speed is slower than those of DEABC and MABC. Further, MABC has the fastest speed at the end and is the first one to attain the true global number. When *D* = 40, ABC and DEABC fall into the global solution. NABC has the fastest speed at the beginning and attains the global optimal solution at the end, but the true global number of NABC is smaller than that of MABC. MABC goes into the global optimal solution at the end, and the speed is faster than that of NABC.

The convergence performance of Sphere function exhibits that various behavior for both dimension sizes. When *D* = 20, ABC converges with the slowest speed, and NABC and DEABC have fastest speed in the first place. Later on, MABC is the first to arrive to the global optimal solution, the second one is DEABC, and the third one is NABC. And ABC locates a local optimal solution. When *D* = 40, ABC also goes into the local optimal. NABC, DEABC, and MABC have the similar speed originally; hereafter MABC has quicker speed than NABC and DEABC. Finally, these three algorithms come to the global optimal solution, but the best one is MABC, the second one is DEABC, and the last one is NABC.

The convergence performances of Schwefel 2.22 and Ackley functions demonstrate that, for both dimension sizes of 20 and 40, ABC, NABC, and DEABC all fall into the global optimal solution, but DEABC converges with the fastest speed originally. Later on, MABC has the fastest speed and locates the global optimal solution at the end.

From Figures 1 [Fig fig2] [Fig fig3] [Fig fig4] [Fig fig5]–6, it can be comprehensively deduced that ABC converges with the slowest speed and tends to a local optimal solution. DEABC and NABC have faster speed than ABC but sometimes enter into local optimal solution. MABC has fastest speed at the end and achieves the really global optimal solution.

### 4.2. Comparison of Mean Value and Standard Deviation

The results of comparing the mean value and standard deviation are shown in Table 4, where the first column shows the benchmark functions, the “*D*” column represents the dimension of function, the “Mean” column contains the average best values of benchmark functions, and the “SD” column shows the standard deviation of the best value.

For six benchmark functions, one can know that when *D* = 20, the mean values of ABC, NABC, DEABC, and MABC are better than those of them when *D* = 40. For SumSquares, Griewank, and Rastrigin functions, it is observed that when *D* = 40, the mean value of NABC has better mean than that of DEABC. On the other hand, in terms of standard deviation, ABC and NABC are less than DEABC for Sphere and Ackley functions. In addition, MABC has better average best values than DEABC, and DEABC has better average best values than ABC and NABC. The least standard deviation of MABC to six test functions with the dimensions *D* = 20 and *D* = 40 is 8.89001*e* − 18, and the standard deviations of MABC are always less than DEABC; meanwhile the standard deviation of DEABC is less than ABC and NABC.

Hence, by the results of six test functions with the dimensions *D* = 20 and *D* = 40, MABC have better results than ABC, NABC, and DEABC. For all test functions, the mean and standard deviation of MABC results are several orders of magnitude better than ABC, NABC, and DEABC, particularly in case of SumSquares, Schwefel 2.22, and Ackley functions. Therefore, MABC has more accurate solution and better stability than ABC, NABC, and DEABC.

### 4.3. Comparison of Total Run Time

**Figure 7:**
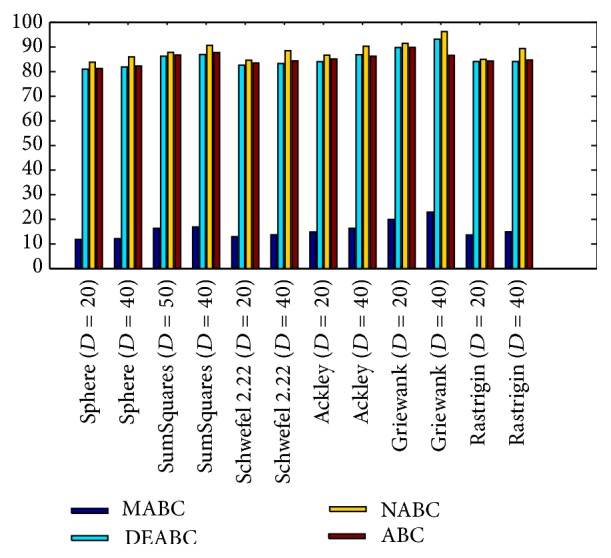
Total run time comparison of ABC, NABC, DEABC, and MABC.

This subsection will show effectively that the searching speed of MABC is fastest through the phenomenon for total run time of solutions (see Figure 7). To all the functions, when *D* = 20 or 40, NABC costs the more time, ABC and DEABC follow closely, MABC spends time the least, and the time of search solution of MABC is independent of dimension sizes. And the Sphere function costs least computational time, while the Rastrigin function takes the maximum one, so it shows that the finding solution time is related to the function types. Further, the time of search solution of MABC is less than that of any one of ABC, NABC, and DEABC.

Moreover, based on the six benchmark functions with the dimension *D* = 20 or 40, Table 5 displays all of the reduction rates, and the reduction rate of total iteration time is at least 73.52% and up to 85.88% for MABC to DEABC, MABC to NABC, and MABC to ABC.

## 5. Concluding Remarks

In this paper, to improve classical artificial bee colony algorithms, a new modified artificial bee colony (MABC) algorithm was developed, which is poor at exploitation and has some defects such as slow search speed, poor population diversity, the stagnation in the working process, and being trapped into the local optimal solution.

In order to make the initial population diversity distribute evenly and to take full use of the search space information, the initial population was constructed by using the opposition-based learning theory. Next, a new *S*-type method of grouping was applied to reduce the difference between each group and replaced a greedy method (i.e., the roulette wheel way) by free search algorithm-sensitivity with pheromone. Then, a suitable exponential function updating length step was designed for replacing the original random step and carrying out faster and more stable searching of the global optimal solution.

Finally, from the new test function versions CEC13, six benchmark functions with the dimensions *D* = 20 and *D* = 40 were chosen and applied in the experiments for analyzing and comparing the iteration speed and accuracy of MABC to differential evolution artificial bee colony algorithm (DEABC), novel artificial bee colony algorithm (NABC), and basic artificial bee colony algorithm (ABC). With respect to the six benchmark functions with the dimensions *D* = 20 and *D* = 40, respectively, one can see from the experimental results in Tables 4 and 5 that the smallest standard deviation of the new modified algorithm is 8.89001*e* − 18, and the reduction rate of total iteration time for the new modified algorithm relative to the other three proposed artificial bee colony algorithms is at least 73.52% and up to 85.88%. Moreover, the experimental results presented in Figures 1 [Fig fig2] [Fig fig3] [Fig fig4] [Fig fig5] [Fig fig6]–7 show that MABC has faster and more stable searching and can increase poor population diversity and avoid falling into the local optimal solutions.

MABC is superior to ABC, NABC, and DEABC, and the feasibility and effectiveness of MABC are very easy to be implemented. However, some other state-of-the-art algorithms may be selected as comparable algorithms, and more complex test functions and more diverse set of benchmark functions may be implemented to validate the benefits of MABC. Thus, the following works as* open questions* will be worth further studying:

- Some applications of MABC in the areas of pattern classification, fuzzy control, nonlinear system modeling, parameter tuning of proportional-integral-derivative controller, and so on.
- Selecting more state-of-the-art algorithms as comparable algorithms.
- Simulating more diverse set of benchmark functions for corresponding swarm intelligence algorithms.
- Implementing more complex functions for validating the benefits of MABC and other corresponding improving swarm intelligence algorithms.

## Figures and Tables

**Algorithm 1:**
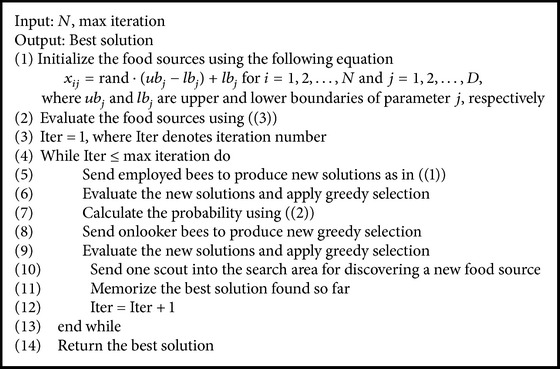
Flowchart of ABC.

**Algorithm 2:**
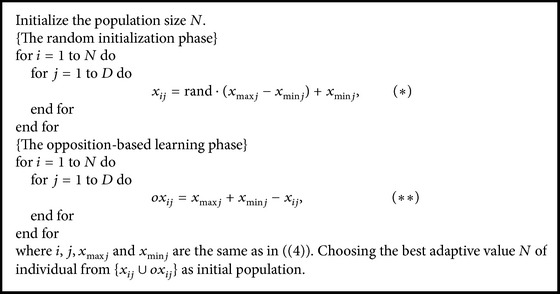


**Algorithm 3:**
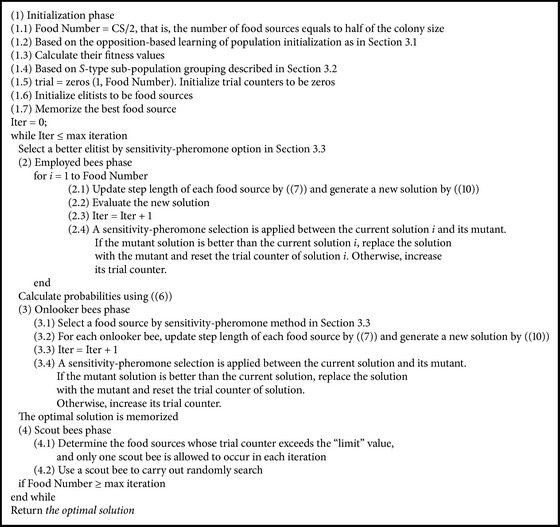
Flowchart of MABC.

**Table 1:** General grouping process.

Solutions	Subpopulations			
	*S*_1_	*S*_2_	⋯	*S*_*M*_
The first round	*Y*_1_	*Y*_2_	⋯	*Y*_*M*_
The second round	*Y*_*M*+1_	*Y*_*M*+2_	⋯	*Y*_2*M*_
⋮	⋮	⋮	⋮	⋮

**Table 2:** *S*-type subpopulation grouping.

Solutions	Subpopulations			
	*S*_1_	*S*_2_	⋯	*S*_*M*_
The first round	*Y*_1_	*Y*_2_	⋯	*Y*_*M*_
The second round	*Y*_2*M*_	*Y*_2*M*−1_	⋯	*Y*_*M*+1_
⋮	⋮	⋮	⋮	⋮
The final round	*Y*_2*nM*_	*Y*_2*nM*−1_	⋯	*Y*_(2*n*−1)*M*+1_
	⋮	*Y*_2*nM*+*K*_	⋯	*Y*_2*nM*+1_

**Table 3:** Numerical benchmark functions.

Name	Formula	Search domain	Minimum
Sphere	$f_{1}\left(x\right)=\overset{D}{\underset{i=1}{\sum }}x_{i}^{2}$	[−100,100]^*D*^	0
SumSquares	$f_{2}\left(x\right)=\overset{D}{\underset{i=1}{\sum }}ix_{i}^{2}$	[−10,10]^*D*^	0
Schwefel 2.22	$f_{3}\left(x\right)=\overset{D}{\underset{i=1}{\sum }}\left|x_{i}\right|+\prod_{i=1}^{D}\left|x_{i}\right|$	[−100,100]^*D*^	0
Griewank	$f_{4}\left(x\right)=\frac{1}{4000}\overset{D}{\underset{i=1}{\sum }}x_{i}^{2}-\prod_{i=1}^{D}\cos\left(\frac{x_{i}}{\sqrt{i}}\right)+1$	[−600,600]^*D*^	0
Rastrigin	$f_{5}\left(x\right)=\overset{D}{\underset{i=1}{\sum }}\left[x_{i}^{2}-10\cos\left(2\pi x_{i}\right)+10\right]$	[−5.12,5.12]^*D*^	0
Ackley	$f_{6}\left(x\right)=-20e^{-0.2\sqrt{(\sum_{i=1}^{D}x_{i}^{2})/D}}$	[−32,32]^*D*^	0
	−*e* ^(∑_*i*=1_^*D*^‍cos⁡(2π*x*_*i*_)⁡)/*D*^ + 20 + *e*		

**Table 4:** Comparison of the mean value and standard deviation.

Function	*D*	ABC		NABC	
		Mean	SD	Mean	SD
Sphere	20	9.12643*e* − 4	6.80695*e* − 4	8.51338*e* − 16	2.29525*e* − 16
	40	0.445472	0.234793	3.7323*e* − 11	1.50446*e* − 12
SumSquares	20	1.3749*e* − 5	2.33632*e* − 5	8.75587*e* − 16	2.30537*e* − 16
	40	0.994557	1.70504	4.65058*e* − 10	2.77676*e* − 10
Schwefel 2.22	20	9.42221*e* − 4	7.46032*e* − 4	2.22277*e* − 8	1.00025*e* − 8
	40	0.0461286	0.0287431	2.15524*e* − 4	6.03173*e* − 5
Griewank	20	2.5264*e* − 6	1.43918*e* − 6	4.44089*e* − 16	1.92296*e* − 16
	40	0.0125412	0.00802599	1.07636*e* − 12	5.37341*e* − 13
Rastrigin	20	0.0331619	0.0574246	7.75912*e* − 14	3.28186*e* − 14
	40	15.5119	20.1043	6.00988*e* − 9	2.57688*e* − 9
Ackley	20	4.22652*e* − 3	3.9353*e* − 3	7.97212*e* − 10	4.51507*e* − 10
	40	0.603485	0.0057116	3.77068*e* − 6	7.14915*e* − 7

Function	*D*	DEABC		MABC	
		Mean	SD	Mean	SD

Sphere	20	5.33457*e* − 16	6.65399*e* − 18	4.21651*e* − 16	8.89001*e* − 18
	40	5.54516*e* − 13	3.68742*e* − 13	1.49866*e* − 15	2.47044*e* − 16
SumSquares	20	6.03584*e* − 16	1.3174*e* − 16	4.16975*e* − 16	1.01262*e* − 16
	40	5.20275*e* − 10	6.16741*e* − 7	3.29277*e* − 15	2.28728*e* − 15
Schwefel 2.22	20	3.61252*e* − 6	1.24222*e* − 6	8.79872*e* − 16	1.09679*e* − 16
	40	1.08779*e* − 4	1.65357*e* − 4	4.12959*e* − 10	1.80491*e* − 10
Griewank	20	3.70074*e* − 16	2.31111*e* − 16	1.11022*e* − 16	0
	40	6.54372*e* − 11	8.03242*e* − 11	8.14164*e* − 16	2.34132*e* − 16
Rastrigin	20	2.84217*e* − 14	2.84217*e* − 14	9.4739*e* − 15	1.64093*e* − 14
	40	4.40958*e* − 7	3.26047*e* − 7	1.89478*e* − 13	3.28186*e* − 14
Ackley	20	1.21723*e* − 6	1.197*e* − 6	3.16784*e* − 14	2.05116*e* − 15
	40	7.67309*e* − 7	5.03943*e* − 7	5.01809*e* − 9	1.85715*e* − 9

**Table 5:** Reduction rate of total iteration time.

Function	*D*	MABC to DEABC	MABC to NABC	MABC to ABC
Sphere	20	85.38%	85.88%	85.43%
	40	85.11%	85.83%	85.19%
SumSquares	20	81.08%	81.41%	81.17%
	40	80.59%	81.38%	80.77%
Schwefel 2.22	20	84.26%	84.64%	84.42%
	40	83.53%	84.50%	83.74%
Griewank	20	77.81%	78.22%	77.83%
	40	75.39%	76.19%	73.52%
Rastrigin	20	83.80%	83.96%	83.84%
	40	82.23%	83.28%	82.35%
Ackley	20	82.26%	82.80%	82.48%
	40	81.20%	81.93%	81.07%
